# A Self-Healing Thermoset Epoxy Modulated by Dynamic Boronic Ester for Powder Coating

**DOI:** 10.3390/polym15193894

**Published:** 2023-09-26

**Authors:** Yongqi Liu, Ziyuan Li, Caifu Zhang, Biru Yang, Hua Ren

**Affiliations:** 1 Ningbo Research Institute, Zhejiang University, Ningbo 315807, China; yongqiliu@zju.edu.cn (Y.L.); biruyang@zju.edu.cn (B.Y.); 2 State Key Laboratory of Chemical Engineering, College of Chemical and Biological Engineering, Zhejiang University, Hangzhou 310027, China; 3 School of Biological and Chemical Engineering, Ningbo Tech University, Ningbo 315100, China; ziyuan-li@outlook.com; 4 Tongling Shanwei New Material Technology Inc. Co., Ltd., Tongling 244000, China; 13855902233@139.com

**Keywords:** self-healing, photo-thermal, boronic ester, epoxy, powder coating

## Abstract

Thermoset powder coatings exhibit distinctive characteristics such as remarkable hardness and exceptional resistance to corrosion. In contrast to conventional paints, powder coatings are environmentally friendly due to the absence of volatile organic compounds (VOCs). However, their irreversible cross-linking structures limit their chain segment mobility, preventing polymers from autonomously repairing cracks. Dynamic cross-linking networks have garnered attention for their remarkable self-healing capabilities, facilitated by rapid internal bond exchange. Herein, we introduce an innovative method for synthesizing thermoset epoxy containing boronic ester moieties which could prolong the life of the powder coating. The epoxy resin system relies on the incorporation of two curing agents: one featuring small-molecule diamines with boronic bonds and the other a modified polyurethane prepolymer. A state of equilibrium in mechanical properties was achieved via precise manipulation of the proportions of these agents, with the epoxy composite exhibiting a fracture stress of 67.95 MPa while maintaining a stable glass transition temperature (Tg) of 51.39 °C. This imparts remarkable self-healing ability to the coating surface, capable of returning to its original state even after undergoing 1000 cycles of rubbing (using 1200-grit abrasive paper). Furthermore, the introduction of carbon nanotube nanoparticles enabled non-contact sequential self-healing. Subsequently, we introduce this method into powder coatings of different materials. Therefore, this work provides a strategy to develop functional interior decoration and ensure its potential for broad-ranging applications, such as aerospace, transportation, and other fields.

## 1. Introduction

Thermoset polymers in chemical industry have garnered widespread attention for their remarkable mechanical properties [1,2] encompassing notable traits such as dimensional stability [3], resistance to creep [4], and chemical corrosion resistance [5]. These distinctive attributes have stimulated their extensive applications in various sectors such as transportation [6] (high-speed rail, aircraft), energy generation [7] (windmill generators, solar cell panels), sporting equipment [8] (bicycles, rackets, golf clubs) and so on. In the field of coatings, powder coatings stand out for their high production efficiency, automated production, energy savings, and environmental safety [9]. Within the realm of powder coatings, thermoset powder coatings constitute a substantial proportion and can be broadly classified into categories such as epoxy powder coatings [10], polyester powder coatings [11], and polyurethane powder coatings [12]. Epoxy powder coatings represent a category of thermoset powder coatings that emerged during the 1960s [13], which boast a diverse array of subtypes and a wide spectrum of applications, demonstrating pronounced efficacy in domains encompassing interior adornment and exterior anti-corrosion [14].

However, for interior decorative epoxy resin powder coating systems, it is inevitable that defects will arise due to inadvertent collisions or friction in routine usage [15]. The inherent irreversible cross-linking network structure of epoxy resins imposes constraints upon the mobility of polymer chain segments. The confinement engenders challenges in eradicating abrasion-induced scratches, resulting in the inability to reinstate the original state or to effectuate restoration for subsequent utilization [16]. Therefore, this phenomenon leads to a substantial curtailment of the service life exhibited by powder coatings.

The self-healing mechanism of polymers involves a responsive reaction to external environmental changes such as light [17,18], temperature [19,20,21], and humidity [22,23], resulting in gradual recovery of the damaged area towards its original state and achieving self-healing. Surface or intrinsic self-healing in materials primarily employs physical and chemical methods. Physical methodologies encompass shape memory effects [24,25,26], microcapsule “core-shell” structures [27,28], micro-phase separation [29], and molecular chain diffusion [30], while chemical approaches involve introducing free radicals [31], covalent bonds, and non-covalent interactions (ionic [32], hydrogen [33], supramolecular interactions [34]). As for covalent bonds, the toolkit includes the Diels–Alder reaction [35,36,37], disulfide bonds [38,39], hindered urea bonds [40,41], and boronic ester bonds [42,43], among others. Polymer networks comprising dynamic covalent bonds exhibit network reversibility. Under external forces, polymer chain segments may incur damage; however, due to the dynamic bond exchange triggered by external stimuli, micro-level self-healing occurs at the damage interface. Leveraging the rapid exchange of dynamic covalent bonds enables chemical restoration of fractured chain segments.

Thermoset resins, such as epoxy and polyester resins, exhibit high cross-linking density; furthermore, they possess higher glass transition temperatures (Tg) [44], thus necessitating high healing temperatures and prolonged healing durations. The imperative challenge currently confronting the research domain revolves around the modulation of polymer mechanical properties and the content of internal dynamic covalent bonds. It aims to balance the mechanical performance and self-healing capacity, which constituting an exigent matter necessitating resolution.

In this study, we introduced boronic ester into the epoxy resin structure, consequently incorporating dynamic covalent bonds into the cross-linking networks. The synthesis of the dynamic networks comprised a two-step procedure. The first step involved the synthesis of curing agents, encompassing the dehydration-driven esterification reaction to yield a small-molecule curing agent named NBN as well as the grafting of boronic ester onto the ends of polyurethane prepolymers in order to obtain a modified curing agent named NHP. The second step entailed the cross-linking of epoxy and curing agents via a “click” ring-open reaction. The amalgamation of these two curing agents involves the fine-tuning of the content of NBN and NHP. This manipulation introduced an increased number of boronic ester bonds while simultaneously preserving the mechanical properties of polymers. At specific ratios, the epoxy resin demonstrated a remarkably elevated self-healing ability and expeditious scratch repair efficacy by underscoring the synergy between the introduced boronic ester bonds and the inherent dynamic covalent interactions.

Furthermore, CNT nanoparticles were introduced into the aforementioned system to investigate the photo-thermal conversion efficiency of photo-thermal fillers at content. Under an 808 nm infrared laser, localized temperature on the surface of samples could significantly surpass its Tg. This distinctive phenomenon facilitated targeted and localized self-healing effects. Ultimately, the above methodology could be extrapolated to the industrial area of powder coatings, imbuing them with the capacity for self-healing and thereby extending their operational lifespan.

## 2. Materials and Experiments

### 2.1. Materials

3-Aminophenylboronic acid monohydrate was bought from Leyan (Beijing, China). 3-Amino-1,2-propanediol, Methanol, 1,6-Diisocyanatohexane (HDI), and Dibutyltin Dilaurate were all purchased from Aladdin (Shanghai, China). Ethoxylated bisphenol A (BPE, Mn = 492) and anhydrous Magnesium sulfate were acquired from Macklin (Shanghai, China). N,N-Dimethylformamide (DMF) was supplied by Meryer (Shanghai, China). Epoxy resin E12 which had an epoxy equivalent weight of approximately 830 g/mol was purchased from Shanfu, Anhui, China. All chemicals were used as received.

### 2.2. Synthesis of Small-Molecule Curing Agent (NBN) and Modified Curing Agent (NHP) Containing Boronic Ester Bonds

**Figure 1:**
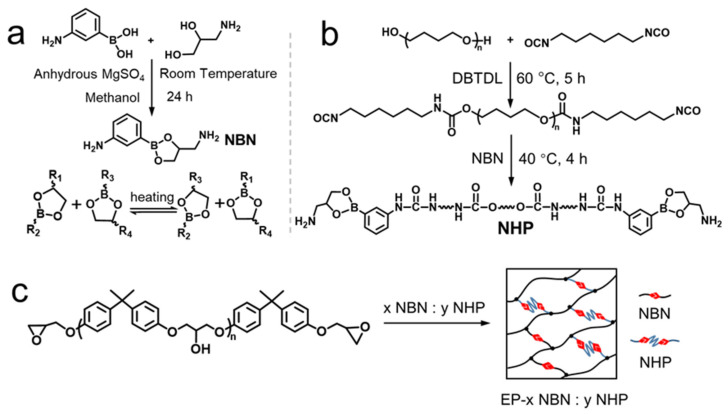
Synthesis procedure and epoxy dynamic networks. (**a**) Esterification reaction of NBN. (**b**) Compound process of NHP. (**c**) The design of dynamic networks with different formulations.

The chemical structures of two curing agents are shown in Figure S1, and the synthesis routines are illustrated in Figure 1a,b.

The first synthesis step was NBN; briefly, anhydrous Magnesium sulfate (10 g, 0.08 mol) and 3-Aminophenylboronic acid monohydrate (13.7 g, 0.1 mol) were mixed in a three-necked flask including 50 mL methanol solvent, followed by the addition of 3-Amino-1,2-propanediol (9.11 g, 0.1 mol) with 25 mL methanol solvent. The mixture was allowed to react at room temperature for 24 h. Afterwards, spin evaporation was carried out at 60 °C for 1 h to remove the methanol solvent. 

Meanwhile, HDI (17.0 g, 0.101 mol), BPE (24.8 g, 0.05 mol), and DMF (40) mL with DBTDL (0.1 wt%) were mixed together at 65 °C for 5 h in order to acquire polyurethane prepolymers. Finally, NBN (19.2, 0.1 mol), polyurethane prepolymers (82.8 g, 0.1 mol), and DMF (60) mL were allowed to react together without catalyst at 45 °C for another 4 h. Afterwards, spin evaporation was carried out at 80 °C for 1 h to remove the DMF solvent.

All products needed to be placed in a vacuum oven at 70 °C overnight to remove solvents. At the end of the reaction, we acquired two kinds of curing agents containing boronic ester bonds.

### 2.3. Synthesis of Dynamic Cross-Linking Networks of Epoxy Resin

Based on the number of active hydrogen and epoxy groups, the molar ratio of curing agents to epoxy resin was 1:2. The content ratios of NBN and NHP were 100%:0, 75%:25%, 50%:50%, 25%:75% and 0:100%, respectively. Afterwards, anhydrous DMF solution (50 wt%) was added to the system. The epoxy resin and curing agents were fully mixed via stirring at 100 °C. A vacuum oven was used to remove the bubbles produced via mechanical mixing. After that, the mixed liquid was placed in the oven at 100 °C, 120 °C, or 140 °C, respectively, and cured for 2 h. Finally, the mixture was placed in the vacuum oven at 70 °C overnight to completely remove the solvent. The thermodynamic properties of polymer networks with different formulations were studied as followed. According to the content of NBN and NHP curing agents in reaction system, five kinds of cross-linking epoxy resin networks could be acquired. They were as follows: EP-100% NBN, EP-75% NBN:25% NHP, EP-50% NBN:50% NHP, EP-25% NBN:75% NHP, EP-100% NHP.

### 2.4. Characterization of Synthesis

All the spectra of ^1^H NMR were tested by Bruker (AVANCE NEO, 400 M, Mannheim, Germany). The deuterated solvent was CHCl_3_.

### 2.5. Characterization of Mechanical and Thermal Property

The dumbbell-shaped samples with a size of 12 × 2 × 1 mm^3^ were prepared in advance. The mechanical properties of samples were determined using a tensile testing machine (Mtssans, E44.104, Shenzhen, China) at a tensile speed of 50 mm/min. The performance measurement results of Tg was provided by DSC (Q800, TA, New Castle, DE, USA), heating rate = 10 °C/min. Thermal stability analysis was evaluated via TGA (Netzch, TG 209 F3, Turnpike Burlington, MA, USA) at a heating rate = 10 °C/min.

### 2.6. Self-Healing Test

Rectangular samples with a size of 5 mm × 40 mm × 1 mm were prepared in advance and cut in half; the two parts were joined with an overlap area of 5 mm × 5 mm and fastened between two glass plates with clips. The whole assembly was put in an oven at different temperatures (80 °C, 100 °C, 120 °C) and heated for different lengths of time (0.25 h, 0.5 h, 1 h, 2 h, and 4 h), respectively.

### 2.7. Photo-Thermal Test

An infrared laser was used as the main source of light-to-heat conversion, and the power of the laser was set at three levels, which were 300 mW/cm^2^, 600 mW/cm^2^, and 900 mW/cm^2^. An infrared camera (Hikvision, Hangzhou, China) monitored changes in surface temperature, and a white light Interferenc microscope measured the width and depth of cracks during self-healing and captured the characteristics of the cracks’ surface topography.

## 3. Result and Discussion

In accordance with the experimental method outlined in Figure 1a,b, the modified curing agent NBN, as well as NHP, was successfully synthesized by grafting NBN onto both terminal moieties of polyurethane prepolymers. Both structures were confirmed via H NMR (Figure S1). Boronic ester bonds are known to exhibit ester bond cleavage reactions at elevated temperatures, even in the absence of water [45]. This phenomenon leads to rapid bond exchange within distinct segments of boronic ester bonds at heterogeneous interfaces within the fractured material. Following complete reaction, recombination takes place at the fracture interface, thereby facilitating a self-healing mechanism [46]. The NHP curing agent features rigid “hard segment” domains encompassing a five-membered ring and a phenyl ring at either extremity, conjoined via a “soft segment” region incorporating constituents such as urethane, urea, and ether linkages. The micro-phase separation is conducive to the movement of molecular chains while maintaining its mechanical properties [47]. Via the introduction of varied proportions of NHP into the reaction formulation, dynamic cross-linking architectures were derived (Figure 1c). The influence of NHP content on the polymer cross-linking density was investigated. As the NHP content increased, the distance between cross-linking points extended due to the elongation of the chain length, resulting in a decrease in the cross-linking density of polymer networks [48]. Moreover, due to the congruence with the number of epoxy-reactive hydrogen moieties, an increase in NHP content results in a proportional rise in boronic ester bonds.

**Figure 2:**
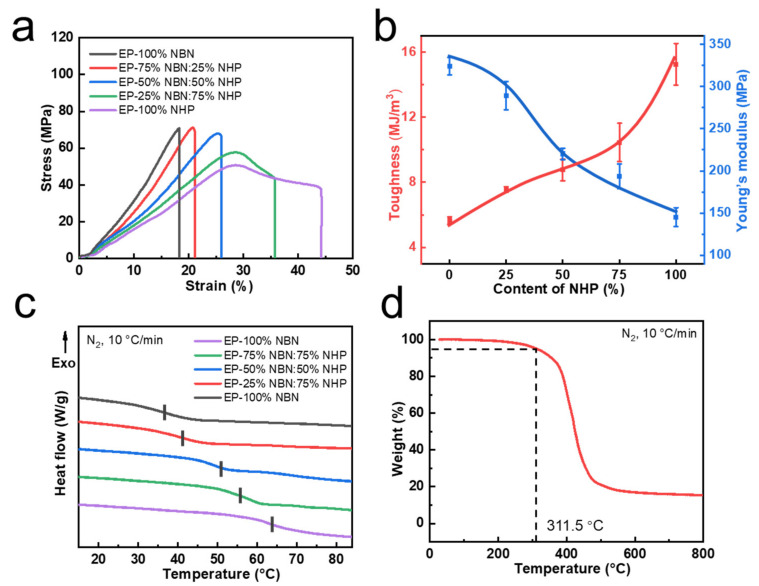
Mechanical and thermal properties of epoxy dynamic networks. (**a**) Strain-stress curves of different formulations. (**b**) The variation trend of toughness and Yong’s modulus. (**c**) DSC curves of different samples. (**d**) TGA curve of samples (50% NHP).

To investigate the performance of cross-linking networks with differing NHP content, a thermodynamic characterization was conducted on five distinct systems. As indicated by the stress–strain curves showed in Figure 2a, with the decrease in cross-linking density, the mechanical strength of polymer decreased gradually and the fracture stress of the materials fell within the range of 50 MPa to 70 MPa. A pronounced reduction in fracture stress was observed when the NHP content surpassed 50%, coupled with an increase in fracture strain from the initial 18% to 45%. The preliminary analysis of the curve profiles suggested a transition from brittle to ductile fracture behaviors [49], consistent with the toughening effect induced via NHP incorporation. In Figure 2b, tensile tests provided insights into the changes in the Young’s modulus and toughness of the materials, demonstrating opposing trends as NHP content increased. The toughness was related to the presence of flexible segments and hydrogen bonding interaction [50] in the material. When polyurethane prepolymers were introduced in greater quantities, strong hydrogen bonding interactions were formed between urethane and urea bonds. This dissipated stress during stretching, increasing the power of the material to overcome fracture [51].

The glass transition temperature (Tg) of the cross-linking networks, adjustable within the range of 35 to 64 °C, was determined using heating differential scanning calorimetry (DSC) measurements with a heating rate of 10 K/min (Figure 2c). The gradual approach of Tg towards room temperature was attributed to the increase in flexible segments within the cross-linking networks due to elevated NHP content [52]. The addition of flexible polyurethane prepolymers reduced the rigidity of the polymer. The introduction of flexible connecting segments also extended the relative distance between the crosslinking points, which reduced the cross-linking density of the polymer. Considering the temperature requirements for routine use of indoor decorative powder coatings, as well as the equilibrium between modulus and toughness, a 50% NHP content was selected as the final formulation for the following investigation. The thermo-gravimetric analysis (TGA) curve (Figure 2d) for 50% NHP indicated a temperature of 311.5 °C corresponding to 5% weight loss, signifying a certain thermal stability of cross-linking networks [53]. As the cross-linking density of polymer decreased, the Tg of polymer showed a downward trend and the temperature of 5 wt% loss also decreased gradually [54,55] (Figure S2 and Table S1).

**Figure 3:**
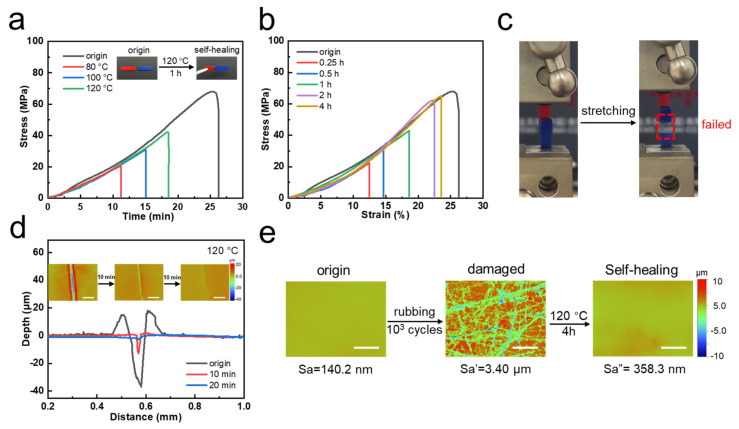
Self-healing performance of dynamic networks containing boronic esters. (**a**) Stress–strain curves of samples under different temperatures and (**b**) different healing times. (**c**) The image of stretching after healing for 4 h at 120 °C. (**d**) Crack comparison during self-healing process. (**e**) Surface roughness after 10^3^ cycles rubbing and self-healing.

Self-healing evaluations were conducted using an “overlap fix” method between two specimens (Figure 3a,b), defining the ratio of fracture stress to initial stress as self-healing efficiency [56]. By maintaining consistent self-healing time/temperature parameters, the impact of these two factors on self-healing efficiency was examined in detail. As temperature increased, molecular chain mobility intensified, leading to ester exchange reactions [57] in the dynamic bonds at the interface, leading to adhesion between the two specimens. As temperature increased, dynamic bond exchanges occurred more rapidly, resulting in higher self-healing efficiency. The self-healing efficiency of the material was directly proportional to the temperature and time of bond exchange; higher temperatures result in faster repair rates within the same timeframe, and longer durations approach the original mechanical properties. Therefore, it is advisable to choose relatively higher temperatures (120 °C) as the repair temperature. Finally, an experimental temperature of 120 °C was selected to evaluate the self-healing performance of polymer materials at various repair durations. As the healing duration increased from 15 min to 4 h, the self-healing efficiency of the material escalated from 32.9% to 95.7%. Figure 3c depicted the appearance of the specimens (healing for 4 h at 120 °C) before and after tension testing, revealing that fracture occurred within the material itself rather than at the overlap. Within the margin of error, these experimental observations suggest that the bonding strength after repairing nearly approached the intrinsic strength of polymer materials, demonstrating effective self-healing effect.

Furthermore, we studied the scratch-healing capacity of the material’s surface at 120 °C using white light interferometry (WLI) to observe the microstructure of the scratched surface (Figure 3d). Combining with profiles of the scratch, observations revealed that after 10 min, the width of the scratch markedly narrowed and the depth decreased by over 50% of the initial value. Afterwards, after a 20-min repair interval, the scratch depth continued to diminish until it nearly disappeared. Scratch self-healing was mainly divided into two specific processes, of which the first step was the adhesion of the scratch interfaces. Due to the increasing temperature of the material matrix, the movement of the polymer chain segment was intensified, which also promoted the leveling of the material surface. When the damaged interfaces came closer, the boronic ester bonds of the adjacent interfaces exchanged dynamically, which further promoted two independent interfaces to form a complete part. Via the common effect of the above two steps, the scratch self-healing effect was finally achieved.

Finally, we simulated damages that could arise during routine usage. WLI was employed to assess surface roughness, denoted as Sa. By subjecting the material surface to repeated abrasion using sandpaper for 1000 cycles, Sa increased from an initial 140.2 nm to 3400 nm. However, owing to abundant dynamic bonds, a notable reduction in Sa was observed after 4 h heating at 120 °C. All the testing methodologies collectively affirm the capability of dynamic covalent bonds to enable self-healing from external damage [58]. Whether it is the overlap of two different interfaces, subtle scratches, random friction or other damage, the dynamic cross-linking system containing boronic ester bonds could effectively respond to external changes and restore the original. This dynamic effect satisfies the ordered or disordered damages produced in our daily use conditions, making the adaptation of the system more extensive. Exploiting this inherent attribute, the development of functional powder coatings with the potential to enhance the service life of coatings would become a promising notion.

**Figure 4:**
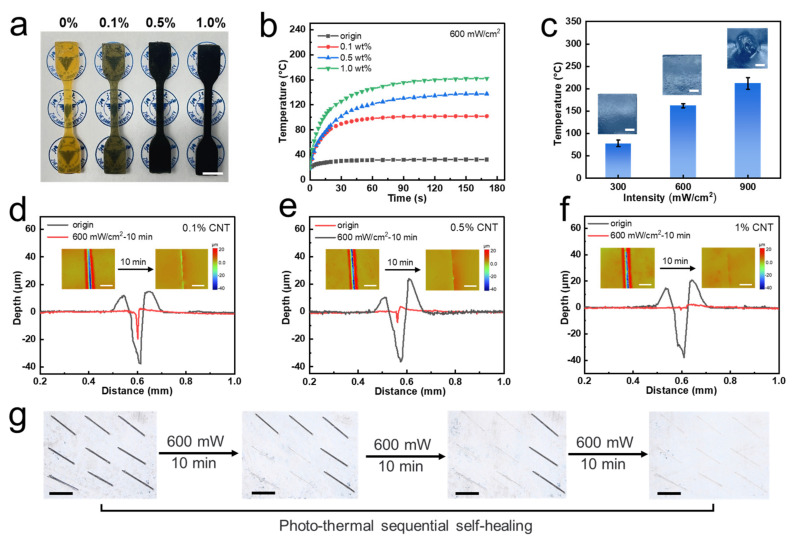
Photo-thermal conversion and its effect in self-healing. (**a**) The image of different CNT contents of samples. (**b**) Time–temperature curves of different samples under 600 mW/cm^2^ infrared laser. (**c**) Max temperature on the surface of samples (1% CNT) under different levels of power. (**d**–**f**) Crack comparison before and after self-healing in different samples under 600 mW/cm^2^. (**g**) Photo-thermal sequential self-healing of 3 × 3 cracks.

Photo-thermal materials are defined as media capable of absorbing specific wavelengths of light and converting light into heat energy and include such examples as polydopamine [59], polypyrrole [60], metal nanoparticles [61], organic dyes [62], and graphene [63]. Moreover, coupling the heat generated via photo-thermal conversion with the self-healing effect of dynamic bonds enables contactless and local self-healing capability. In this work, diverse quantities of multi-walled carbon nanotubes (CNTs), a type of photo-thermal material, were incorporated into the polymer networks (Figure 4a). The effects of CNT content and light intensity on photo-thermal conversion efficiency were explored. As illustrated in Figure 4b and Figure S3, the material surface achieved its maximum temperature as infrared laser intensity and CNT content increased. However, at 1 wt% CNT content and 900 mW intensity, the material underwent melting due to excessive temperature, leading to morphological destruction on the surface (Figure 4c). Simultaneously, pre-made scratches were introduced on materials with varying CNT contents, followed by 10 min of photo-thermal self-healing process via laser irradiation. The scratch changes were monitored using WLI (Figure 4d–f). A discernible trend emerged: the scratches exhibited shallower depths after healing when a greater quantity of photo-thermal fillers was incorporated. With 1 wt% CNTs content, the scratch almost disappeared. Given the thermal tolerance limitations, the experimental conditions were eventually set at a CNT content of 1 wt% and light intensity of 600 mW.

As depicted in Figure 4g, a 3 × 3 array of pre-made scratches was introduced on the polymer surface. Laser irradiation was first applied to the lower left four scratches. Microscopic imagery revealed that the scratches within the designated area had vanished, while the rest of the area retained its original morphology. Subsequently, localized irradiation was sequentially administered to the upper left and rightmost column, producing the self-healing effect exclusively within the targeted regions. The scratch configuration transformed from a numeral “7” to a “1” and eventually disappeared. This phenomenon underscored that the utilization of photo-thermal conversion could achieve spatially continuous self-healing effect. Resin containing photo-thermal fillers has the characteristics of non-contact and fixed-point self-healing ability, which could be well applied to the powder coating field. When powder coatings are locally damaged, it is not necessary to put the whole coating into the oven for heating at a high temperature. Using local laser irradiation by itself could achieve the self-healing effect. This measure avoids the large volume of coatings, the inconvenient environment, and other complex factors. It would not only not need to be placed in a large oven, but would also reduce energy consumption, leading to economic benefits.

**Figure 5:**
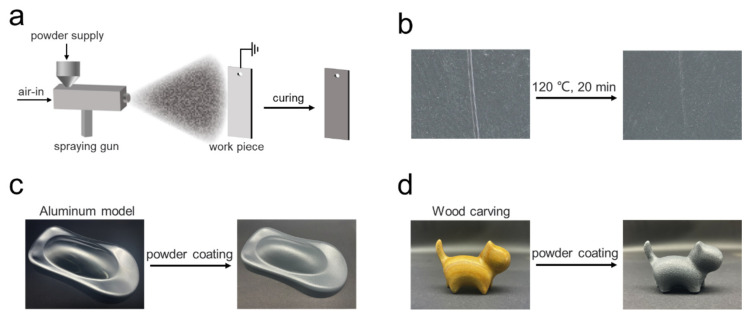
Application of self-healing epoxy system in powder coating. (**a**) Diagram of electrostatic powder spraying. (**b**) Self-healing ability of functional powder coating. (**c**) Powder coating on aluminum model. (**d**) Powder coating on wood carving.

In the end, a novel composition for a functional powder coating was formulated by blending the base resin with NBN and NHP curing agents, along with additives such as fillers, leveling agents, and defoamers, including 57.1% E12, 1.7% NBN, 15.4% NHP, 20.5% TiO_2_ filler, 1.4% SiO_2_ adsorbent, 1.1% leveling agent, 0.5% 701B brightening agent, 0.6% 5031 brightening agent, and 0.6% benzoin defoaming agent, following a sequence of processing steps, including grinding, extrusion, cooling, fracturing, and electrostatic spraying [64] (Figure 5a and Figure S4). The curing temperature obtained for the laboratory formulation was 140 °C for 30 min. The particle size after grinding and sieving was 160 mesh. The electrostatic spray voltage ranged from 50 to 70 kV, and the spraying distance was approximately 15 to 20 cm. We achieved the production of sample boards coated with the self-healing powder coating. When samples incurred scratches on surface, the scratches disappeared immediately upon exposure to hot air from a hair dryer (Figure 5b). What is more, this self-healing coating could be applied to complex three-dimensional structures of various materials, such as aluminum models and wooden carvings (Figure 5c,d), thereby enhancing the aesthetic appeal of the coatings while extending their lifespan. The incorporation of a small amount of curing agent imparted self-healing capabilities to the coatings, thus reducing expenses and energy consumption to a certain extent. This functional coating not only provided protective and isolating attributes to the materials but also endowed them with surface self-healing capability.

## 4. Conclusions

In summary, a kind of functional material with good thermal stability and self-healing capacity was obtained. We effectively synthesized boronic curing agents for curing epoxy at an appropriate ratio to introduce dynamic covalent bonds into cross-linking networks. In addition, the introduction of photo-thermal fillers into the epoxy system could generate the healing temperature on the material surface quickly. Via infrared laser irradiation, the sequential self-repair of scratches could be realized. By applying the experimental formulas to the production process of epoxy powder coating, we successfully developed a self-healing powder coating. The formulation of 50% NBN:50% NHP generated rapid scratch repair at 120 °C for 20 min. By incorporating a 1% content of CNT, the surface temperature could reach 160 °C within 120 s, far surpassing the range of single thermal repairing, enabling rapid scratch repairing within 10 min. This functional powder coating achieved remarkable performance in functional interior decoration and is expected to be applied in aerospace, transportation, and other fields.

## Data Availability

The data presented in this study are available on request from the corresponding author.

## Supplementary Materials

The following supporting information can be downloaded at: https://www.mdpi.com/article/10.3390/polym15193894/s1, Figure S1: Chemical structure and the 1HNMR spectra of (a) NBN and (b) NHP; Figure S2: The thermogravimetric analysis (TGA) curves for all resins under nitrogen atmosphere, the heating rate = 10 °C/min; Table S1: Thermal stability factors of five samples obtained from TGA and DTG curves; Figure S3: Time-temperature curves of different samples under different power of infrared laser. (a) 300 mW/cm^2^, (b) 600 mW/cm^2^, (c) 900 mW/cm^2^; Figure S4: The imagines of powder coating process, (a) premixing and grinding, (b) melt extrusion, (c) cooling fracturing, (d) screening and grading, (e) electrostatic spraying, (f) curing and film forming.
