# Supplementary material for: A Self-Healing Thermoset Epoxy Modulated by Dynamic Boronic Ester for Powder Coating
Source: Polymers (Basel). 2023 Sep 26;15(19):3894. doi: 10.3390/polym15193894 (PMC10575017; doi:10.3390/polym15193894)
Supplement: Supplementary file 1 [file polymers-15-03894-s001.zip › polymers-2614482-supplementary.pdf]

A self-healing thermoset epoxy modulated by dynamic boronic ester for powder coating

Yongqi Liu[1,2], Ziyuan Li[3], Caifu Zhang[4], Biru Yang[1,2], Hua Ren[1,2*]

1 Ningbo Research Institute, Zhejiang University, Ningbo 315807, China

2 State Key Laboratory of Chemical Engineering, College of Chemical and Biological Engineering, Zhejiang University, Hangzhou 310027,China

3 School of Biological and Chemical Engineering, Ningbo Tech University, Ningbo, 315100, China

4 Tongling Shanwei New Material Technology Inc. Co., Ltd. Tongling, 244000, China

*Correspondence: renh@zju.edu.cn

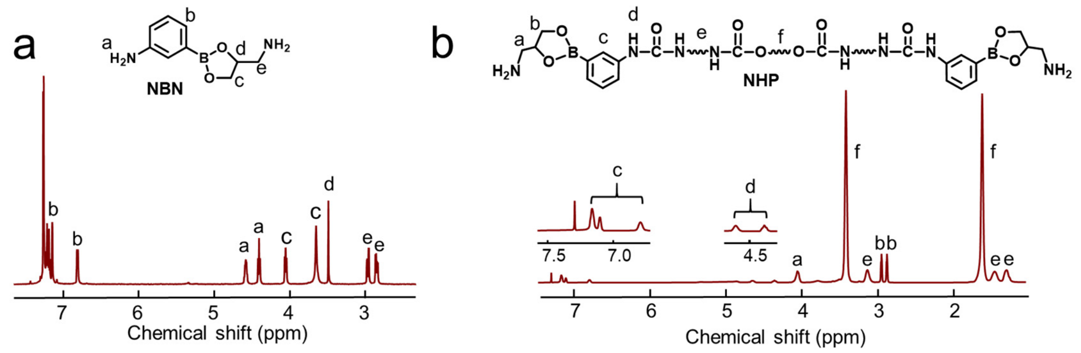

Figure S1. Chemical structure and the 1HNMR spectra of (a) NBN and (b) NHP.

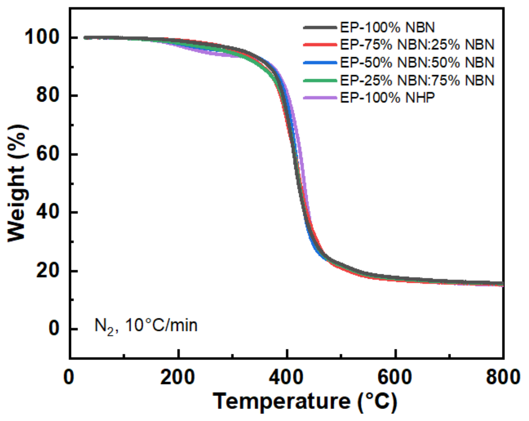

Figure S2. The thermogravimetric analysis (TGA) curves for all resins under nitrogen atmosphere, the heating rate=10 °C/min.

Table S1. Thermal stability factors of five samples obtained from TGA and DTG curves.

Sample	Tg (°C)	$T_{5\ wt\%\ loss}$ (°C)
EP-100% NBN	35.52	326.03
EP-75% NBN:25% NHP	41.70	314.23
EP-50% NBN:50% NHP	51.39	311.84
EP-25% NBN:75% NHP	56.56	295.36
EP-100% NHP	63.71	250.43

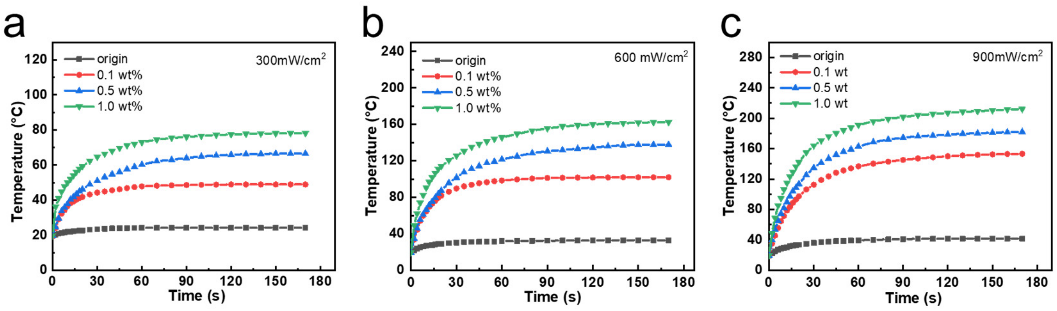

Figure S3. Time-temperature curve of different samples under different power of infrared laser. (a) 300 mW/cm^2, (b) 600 mW/cm^2, (c) 900 mW/cm^2.

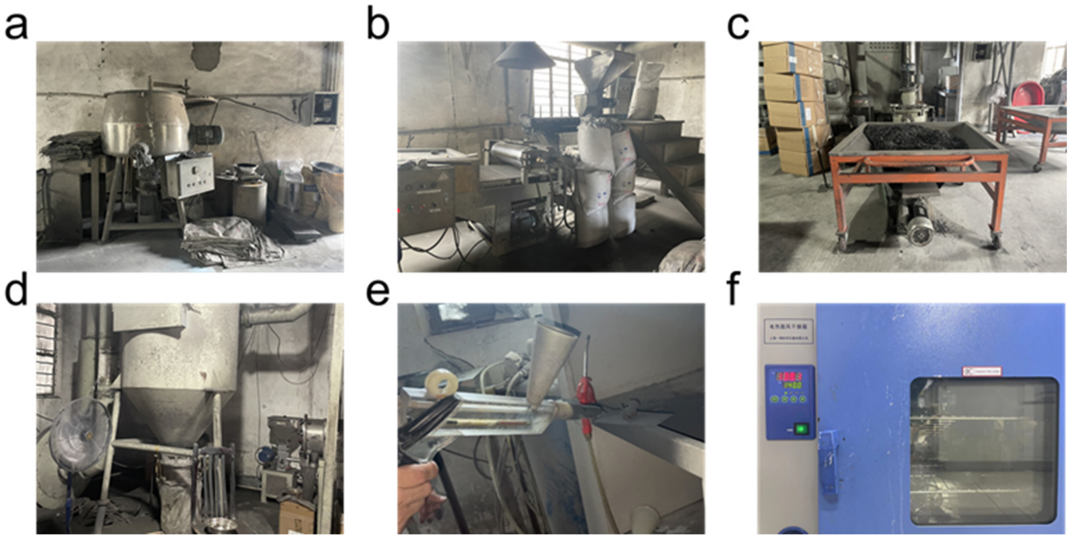

Figure S4. The imagines of powder coating process, (a) premixing and grinding, (b) melt extrusion, (c) cooling fracturing, (d) screening and grading, (e) electrostatic spraying, (f) curing and film forming.
